# COVID‐19, state (in)visibility and structural violence in low‐ and middle‐income countries

**DOI:** 10.1111/issj.12358

**Published:** 2022-07-26

**Authors:** Kate Pincock, Nicola Jones, Khadija Mitu, Silvia Guglielmi, Abreham Iyasu

**Affiliations:** ^1^ Gender and Adolescence Global Evidence (GAGE), ODI London UK; ^2^ Department of Anthropology University of Chittagong Bangladesh; ^3^ GAGE Ethiopia Addis Ababa Ethiopia

## Abstract

The socioeconomic impact of COVID‐19 on adolescents and youth in lower‐ and middle‐income countries (LMICs) who have migrated for work, are among the urban poor, or have been forcibly displaced is not well understood. To address this knowledge gap, this article draws on in‐depth qualitative interviews undertaken between April and July 2020 with 249 adolescent girls and boys and 24 community key informants in Bangladesh and Ethiopia. These two countries have divergent social protection systems and thus provide a useful comparative lens to understand state provisioning for the most disadvantaged, including vulnerable young people, in crisis contexts.

Despite rapid implementation of restrictions to halt viral spread, the mobilisation of social protection in response to the pandemic's socioeconomic effects has lagged. Using a lens of structural violence, findings underscore that socially marginalised young people are the most disadvantaged by state failures to deliver essential services or protection. There has also been insufficient support from humanitarian and development actors in responding to the challenges of the pandemic. The article concludes that identifying and addressing how structural inequalities shape access to and inclusion in social protection mechanisms can contribute to more effectively targeted measures to support the most disadvantaged, especially during crises.

## 1 INTRODUCTION

The emergence and spread of the novel coronavirus COVID‐19 in early 2020 has presented states around the world with hitherto unseen governance challenges. In low‐ and middle‐income countries (LMICs), strategies to manage the pandemic have included lockdowns, social distancing, work‐from‐home orders, entire non‐essential business sector shutdowns, and closure of schools. Yet in countries where governments are facing existing challenges around political legitimacy and economic stability, the pandemic has generated further cracks in state–citizen relations. Both Ethiopia and Bangladesh are large and populous countries, known for large‐scale poverty reduction programming targeting the very poorest, which has historically attracted significant donor support (Islam 2016; Lavers 2019). Ethiopia was initially seen as a success story in its quick response to the pandemic, but has since seen transmission rates rise rapidly, with a six‐fold increase in recorded cases between June and August 2020 (UNOCHA 2020b). By March 2021 the country had recorded over 188,000 cases and 2,674 deaths (Dong et al. 2022). Meanwhile, in Bangladesh, the government was slow to deploy testing or to shut down businesses, leading to a rapid spread of the virus very early on (Chowdhury, Sunna, and Sanjoy 2020). While Bangladesh began to roll out its vaccine programme in January 2021, by March 2021 the country had recorded 573,687 cases and 8,720 deaths (Dong et al. 2022).

Notwithstanding that COVID‐19 primarily affects older people and those with existing health conditions, there has been widespread concern about the social and economic impact of the pandemic on adolescents and young people (OHCHR 2020) given that previous crises – the 2008 global financial crisis, the Ebola epidemic in West Africa, and the AIDS pandemic – resulted in heightened inequalities (Antonopoulos 2009; Jones and Marsden 2010; Onyango et al. 2019; Razavi et al. 2020). Ethiopia and Bangladesh both face existing governance challenges. In Ethiopia, many young people migrate from rural areas to urban centres for work, and prior to the pandemic, the country was dealing with high rates of youth urban unemployment (Atnafu, Oucho, and Zeitlyn 2014; Grabska, DE Regt, and Del Franco 2018). In Bangladesh, a significant proportion of the population continue to live in extreme poverty, and generations continue to be politically and economically marginalised (Zulfiqar, Mujeri, and Badrun Nessa 2014). Both countries also host large numbers of displaced people, of whom around half are under 18 and lack basic rights, as well as facing disruptions to their education (UNHCR 2018; Wanjiru 2018; Jones, Yadete, and Pincock 2019a) and challenges in securing safe work (Evans, Lo Forte, and Mcaslan Fraser 2013; Gercama et al. 2018; Guglielmi et al. 2019; Jones, Devonald, and Guglielmi 2019b).

The 2030 Agenda and the Sustainable Development Goals (SDGs) promote transformational governance strategies to foster resilience based around values such as equity, inclusion, accountability, justice and respect for diversity (UNDESA 2020). To meet the governance objectives of Agenda 2030 to “leave no one behind”, states must be effective in protecting the poorest and most vulnerable during times of crises and provide essential services for all (UNDESA 2020). In politically fragile contexts, the World Bank (2017) has also called for governance to be placed at the centre of development assistance.

Given these objectives, this paper asks: how can states protect vulnerable young citizens and deliver on assistance in the context of an emergency such as COVID‐19? And what are the implications for legitimacy when states fail to do so? To answer these questions, research was undertaken with young people in Ethiopia and Bangladesh exploring their experiences of the pandemic and their perceptions of the challenges it presents. This research was undertaken as part of a broader longitudinal international research programme, Gender and Adolescence: Global Evidence (GAGE), which seeks to establish “what works” in expanding the capabilities of adolescents in LMICs. Their experiences of structural violence during the pandemic, at the level of the household and community and in their encounters with the state, are the focus of this paper.

## 2 EFFECTIVE GOVERNANCE DURING CRISES

Recent years have seen increasing attention to effective governance as a key component of development. SDG 16 identifies “the development of just, peaceful and inclusive societies” as a key objective for Agenda 2030, and in 2018, basic principles for effective governance were endorsed by the United Nations Economic and Social Council (Committee of Experts on Public Administration 2018). These principles include the commitment to leave no one behind and promote intergenerational equity and participation – which are vital for young people's inclusion in the governance agenda. The World Development Report 2017, *Governance and the Law*, asks how states can design and implement policies that improve citizens’ lives, describing effective governance as being driven by commitment, coordination, and cooperation (World Bank 2017).

The World Development Report argues that good governance is not about ensuring sustained periods of growth and stability, but rather being able to effectively weather crises (World Bank 2017). States that have implemented strategies for effective governance can adapt and intervene when a crisis happens, whereas states that have not consistently made themselves accountable to citizens and delivered equitable and inclusive policies within stable and transparent conditions will inevitably struggle to secure commitment, coordination, or cooperation.

A lack of cooperation, commitment, and coordination presents challenges for effective governance during crises. A body of work explores the role of the state in protecting vulnerable citizens in times of rapidly emerging crisis such as virus outbreaks or “natural” disasters that require quick action by governments (O'sullivan and Bourgoin 2010; Egger et al. 2020; Vinck et al. 2019; Verwimp, Justino and Brück 2019). Egger et al. (2020) argue that it is essential that there is trust that states know what they are doing and will take measures to protect those adversely affected in order to secure citizen compliance. During the recent Ebola outbreak in the Democratic Republic of Congo, Vinck et al. (2019) show that distrust of local authorities was associated with decreased likelihood of adopting preventive behaviours and seeking treatment. There has been some research attention to the risk of this resulting in conflict within communities (Verwimp, Justino, and Brück 2019) and concerns that lockdowns may erode public trust and exacerbate conflicts in countries with a history of civil unrest (Egger et al. 2020).

The World Bank's warning about governance challenges in crisis contexts is particularly pertinent for states such as Ethiopia and Bangladesh. Prior to COVID‐19, both were dealing with significant governance challenges, including large, displaced populations. Research shows that epidemics heighten existing inequalities because they disproportionately affect those who are already vulnerable (De Bouchout and De Neubourg 2015; Wilkinson and Leach 2015; Tirivayi et al. 2020). This suggests a need to design social protection measures that account for these vulnerabilities early on (O'sullivan and Bourgoin, 2010). Increased violence against women and children was reported during both the 2013 Ebola outbreaks in West Africa (Onyango et al. 2019) and in the wake of major financial crises (Antonopoulos 2009; Jones and Marsden 2010) due to the impact on livelihoods and gender roles within households. COVID‐19 is also predicted to significantly increase the numbers of people living in poverty globally due to major economic contractions and associated job losses for millions of people (ILO 2020).

As the pandemic continues to evolve, research is drawing attention to the important role of social protection in mitigating the impact of the pandemic for the most vulnerable (see Gerard, Imbert, and Orkin 2020; Gentilini et al. 2020; Razavi et al. 2020). In a systematic review of social protection programming, Abdoul‐Azize and El Gamil (2021) find that governments in LMICs predominantly implemented programmes targeting the poorest and most vulnerable citizens – unlike high‐income countries, which focused on stabilising macroeconomic impacts, and in contrast to previous pandemics, where international institutions played a more significant role. Emergency cash transfers have been the most widely used form of assistance (Gentilini et al., 2020). Abdoul‐Azize and El Gamil (2021), however, call attention to the absence of a comprehensive package of social protection measures aimed at tackling longer‐term risks and vulnerabilities. Indeed, where the economic impacts of lockdowns and other preventive measures have been acknowledged within guidance from international institutions, it is often assumed that the state and private sector can be mobilised to support responses. For example, the World Health Organization (WHO) has asked businesses to protect jobs and livelihoods as well as clients and participate in the international response through production, contributions, and repurposing of production facilities (WHO, 2020a). However, the most vulnerable people, who work for daily wages or with no contracts, are less likely to have access to social protection that will enable them to weather the economic impacts of the pandemic. Without modifying available schemes, their expansion will exacerbate exclusion and poverty and put young people at greater risk (Tirivayi et al. 2020). Indeed, the response to previous disease outbreaks in LMICs has traditionally focused on strengthening health systems and training personnel rather than strengthening social protection and assistance measures (Kelly, 2020). Yet for young people, the socioeconomic consequences of government strategies to contain the pandemic are likely to be far more damaging than its direct health effects.

## 3 STRUCTURAL VIOLENCE AND INVISIBLE POWER

In Bangladesh and Ethiopia, governance responses to COVID‐19 can only be fully understood in relation to the broader context of state failures to commit, coordinate, and cooperate to produce inclusive and transformative development policies. Following Mignolo (2007), action (and inaction) by both states to address the consequences of the pandemic for marginalised youth must in turn be contextualised within global socioeconomic and political relations and histories in which the logics of coloniality and development are inextricably connected. As Harman et al. (2021) observe during the pandemic, global policy leverage and neo‐colonial negotiating power by wealthy countries has led to only partial and temporary fixes being offered to LMICs in dealing with the pandemic, creating a vaccine apartheid whilst the root causes of global disparities remain unaddressed.

Literature on socioeconomic and political inequalities elucidates the dynamics which shape how individuals and communities interact with and respond to the state, with implications for state legitimacy during crises (Galtung 1969; Lukes 2005; Gaventa 2006; Mehta 2016). Lukes (2005) explores how powerful actors secure willing compliance from those whom they dominate. Power is exercised in three dimensions, with the third “face of power” rooted in social norms and deeply embedded structures that allow certain interests within society to dominate precisely because they are almost impossible to identify or disentangle from people's own preferences. This is where the idea of structural violence is useful and complementary; structural violence “shows up as a lack of power” (Galtung 1969, p.171).

Structural violence is theorised as the forms of violence embedded within the structures, histories, ideologies, and institutions to which Lukes points, which in Ethiopia and Bangladesh have been shaped by colonialism and exploitation. Unlike personal violence – which is self‐evident through actions in much the same way that the explicit exercise of Lukes’ “first face of power” refers to – structural violence is pervasive and invisible (Galtung 1969). In her work on inequalities in access to water in Ethiopia, Mehta (2016) draws on work by Lukes (2005) and Gaventa (2006) to explore how power operates in a context of structural violence. Mehta argues that the “normalisation” of certain hegemonic arrangements around resource allocation is problematic because it reframes development challenges as technical issues that can be resolved through universalisable solutions, rather than “the specific outcome of particular forms of structure and power” in a given context. The concept of structural violence has also been used to analyse governance failures associated with the Ebola epidemic in West Africa. Wilkinson and Leach (2015) argue that in Sierra Leone, the crisis was a product of entrenched economic, social, and political injustices – structural violence – which fed into the weaknesses of global health governance in dealing with the outbreak. In her work on post‐disaster recovery programmes, Older (2019) describes a “secondary hazard” of crisis response: inadequate strategies can trigger antagonism between governments and citizens, exacerbating social fragmentation in affected communities, in part due to the desire for accountability and to find someone to blame.

Thinking through the challenges of promoting inclusion, Gaventa (2006) draws on Lukes (2005) to elucidate how power shapes the spaces of civic and political engagement people are able to access. While “visible” power can be equated with observable decision‐making and hidden power is about setting agendas, Gaventa argues that “invisible” power shapes people's expectations of what is acceptable. While the 2017 World Development Report on governance draws attention to power relations, it suggests that it is through institutional reforms that underlying power structures can be reformulated to produce more egalitarian outcomes (World Bank 2017, p.80). The argument that institutional reforms can address marginalisation has meanwhile been challenged by postcolonial scholarship, which draws attention to structural inequalities that are embedded and sustained *through* development policies and processes which undermine the capacity and resources of states to deliver comprehensive social protection programming (Kapoor 2008; Hickel 2017a). Yet even within these matrices of power, understanding how power relations play out in different contexts is key to mobilising social protection and assistance through those modes, spaces, and means that meet the needs of the most marginalised social groups.

## 4 METHODOLOGY

Exploring the experiences of young people in these contexts during the pandemic allows us to present the perceptions of those whose voices are usually marginalised in research on governance and accountability; it also enables us to draw comparisons between different state responses and the impact on vulnerable populations. The GAGE methodological toolkit was designed to elicit the voices and experiences of marginalised adolescent girls and boys (Jones et al. 2019c). It does this through a range of tools, including object‐based interviews, participatory activities, life history timelines, and intergenerational dialogue. The toolkit aims to create space and opportunity for young people to express themselves on their own terms, as well as explicitly engaging with the broader relations of power that shape their lives (Pincock and Jones 2020).

Innovative in‐depth qualitative interviews (Małachowska et al. 2020) were carried out by phone between April and July 2020 with 249 adolescent girls and boys, and 24 community key informants (from the education, health, social protection, and women's and children's sectors). More details of the sample characteristics are provided in Table 1. Interviews were carried out in local languages by in‐country researchers who had built up prior relationships with participants during GAGE's longitudinal research. Interviews were transcribed, translated, and coded using a thematic code book in MAXQDA. Ethics approvals were secured locally and internationally.

**TABLE 1:** Qualitative research sample for Bangladesh and Ethiopia

	**Young cohort girls**	**Young cohort boys**	**Old cohort girls**	**Old cohort boys**				
**Research location**	**(age 10–14)**	**(age 10–14)**	**(age 15–19)**	**(age 15–19)**	(Married girls)	(Adolescents with disabilities)	**Total adolescents**	**Key informants**
BANGLADESH (Dhaka)	3	2	17	8	*6*	*5*	**30**	**4**
BANGLADESH (Rohingya)	6	6	11	7	*6*	*4*	**30**	**7**
ETHIOPIA (Dire Dawa city)	12	9	12	12	*6*		**45**	
ETHIOPIA (South Gondar, Amhara)	12	9	14	14	*6*	* 5*	**49**	
ETHIOPIA (East Hararghe, Oromia)	12	9	14	14	*6*	* 5*	**49**	
ETHIOPIA (Zone 5, Afar)	10	9	13	14	*6*		**46**	**13**
**TOTAL**	**55**	**44**	**81**	**69**			**249**	**24**

## 5 BACKGROUND AND CONTEXT

Bangladesh and Ethiopia represent two complex LMIC contexts where the multi‐dimensional consequences of COVID‐19 and the resulting public health response are layered over interconnected pre‐existing challenges. These include significant governance and security deficits, mass displacement, and growing levels of economic inequality.

### 5.1 Bangladesh

After the first case of COVID‐19 was detected, the government closed schools and encouraged businesses to move online. In May 2020, the Ministry of Labour prohibited factories from laying off workers during Eid celebrations, and stated that workers should receive 65 per cent of payments for April when they were unable to work (KPMG 2020). At the same time, there were concerns around the quality and safety of health care provision. Amnesty International (2020) has reported shortfalls in surgical equipment and patients being turned away from hospitals by staff for fear of catching the virus. The government has taken a reactionary response to criticism of its COVID‐19 measures, arresting people under the 2018 Digital Security Act (Reporters Without Borders 2020). In May 2020, the government approved the disbursement of cash aid for 5 million poor families (representing 3 per cent of the population) (KPMG 2020). It also allocated six months’ worth of food aid for families already enrolled in safety net programmes (Islam and Divadkar 2020). However, existing programming largely overlooks the urban extreme poor due to their lack of documentation and difficulties in verifying income, despite this group being at greatest risk due to overcrowded living conditions and poor sanitation (Sakamoto et al. 2020; Rashid et al. 2020).

Social exclusion and structural violence are perpetuated by the distance between governance structures and communities’ needs. Informal workers – of whom there are more than 50 million in Bangladesh, most of them women – are particularly vulnerable to economic shocks (Mujeri 2019). In Cox's Bazaar, the economic impacts of COVID‐19 on household purchasing power have fuelled tensions between Rohingya refugees and host communities. This has prompted some humanitarian agencies to include Bangladeshi nationals in programming for cash distribution and food rations (Inter‐Sector Coordination Group 2020), emphasising the state's failure to respond effectively to its citizens’ needs.

### 5.2 Ethiopia

Following border and school closures and a work‐from‐home order for public employees in March 2020, the government announced a state of emergency in April. Social distancing (2 metres) was encouraged, and an order issued to reduce public transport capacity by 50 per cent. Many private businesses closed (either partially or fully), and following a surge in prices of essential commodities, the government introduced price controls (Chen 2020). The International Monetary Fund has downgraded its growth forecast for Ethiopia by almost half, and the government's Job Creation Commission estimates that 1.4 million workers will be laid off or otherwise affected by the pandemic (Ethiopia News Agency 2020). Although Ethiopia has the second largest social protection safety net in sub‐Saharan Africa, the Productive Safety Net Programme (PSNP), which usually reaches 8 million food‐insecure households, measures to extend the PSNP to meet the needs of the many people affected by workplace closures due to COVID‐19, or of the most vulnerable groups, including the families of children and adolescents with disabilities, have yet to be rolled out at scale despite a huge rise in the numbers of people needing emergency food assistance (UNOCHA 2020a).

Civil unrest in Ethiopia's Oromia region in July 2020 displaced 9,000 people, making testing and tracing efforts even more difficult (UNOCHA 2020a). National internet outages instigated by the government in response to civil unrest created further problems for organisations trying to coordinate humanitarian assistance (UNOCHA 2020a). Elections scheduled for August 2020 were postponed until early 2021. While the new government under President Abiy Ahmed has sought to reconcile the historical ethnic divisions that triggered this violence, there are ongoing problems in both securing cooperation to end conflict and providing essential services to displaced populations.

### 5.3 Findings

Our research in Bangladesh and Ethiopia finds pervasive levels of structural violence perpetrated by government and humanitarian actors. This not only has significant effects on vulnerable young people's experiences during the pandemic but is also likely to have spillover effects in the future. Evidence of three key clusters of structural violence during the pandemic response were found: (1) inadequate protection against exposure to violence in the home and community; (2) inadequate delivery of essential services, including education, health, and psychosocial support; and (3) failure to ensure adequate livelihood opportunities in the context of deteriorating employment and to provide timely social protection support.

1. Inadequate protection against exposure to violence in the home and community

In both countries, young people reported heightened household stress and tensions due to lockdowns and the ensuing economic downturn. While the phone‐based research methodology may have led to some under‐reporting, a number of young people reported experiencing physical or verbal violence due to these changing household dynamics. As a 17‐year‐old girl from Ethiopia noted: “*Since the coronavirus, mother is spending the whole day at home and she saw everything I do. If I made a mistake, she could insult or beat me*”. Key informants confirmed these reports of domestic violence, as an expert from the Bureau of Women, Children, and Youth Affairs explained:

A major impact of COVID on these [adolescent] age groups is their being highly vulnerable to age‐ and gender‐based violence. For instance, children (females) are raped by their close relatives, including fathers and uncles. There are also young women who are currently more vulnerable to domestic violence as a result of COVID.

In Dhaka (Bangladesh), our interviews found that adolescents had a newfound vulnerability related to police brutality during lockdown enforcement. Adolescents (boys and girls) were fearful of the police, which compelled them to stay indoors and wear masks, since they would be fined or beaten for not doing so, particularly early on. As a 17‐year‐old girl explained: *“The police are catching people. So, I am scared”*.

Similarly, Rohingya refugee adolescents in Cox's Bazar district expressed fears about security forces’ strict enforcement of lockdown. As a 15‐year‐old boy said:

If someone is found outside, he/she will be punished… the government's people would beat him up… the army don't beat the elders, but they can hit us. That's why we always stay at home.

Rohingya girls reported that their mobility was already severely restricted by conservative social norms, which meant that they were largely home‐bound prior to the pandemic. As a result, they tended to talk about fears of police violence more generally or about the experiences of male family members. An 18‐year‐old married girl explained:

[I didn't go out of home earlier and] I don't go out now either. [My husband] goes to the nearest shop and has conversations with people sometimes. He can't go to the big bazar of the camp. There are soldiers there… They hit people if they see anyone in the big bazar.

Interviews with street‐connected young people in urban Ethiopia, a number of whom reported being newly street‐connected due to the sharp rise in unemployment since the pandemic, echoed reports of police brutality. As a 19‐year‐old adolescent girl noted:

They beat us – girls on the streets – until we develop scars. They beat you everywhere… eyes, teeth, legs… They take 500 to 800 birr to release you.

Similarly, an 18‐year‐old girl explained:

I have been​ arrested multiple times. I have been beaten by the police… Mostly, we wear plastic bags and sleep inside the ditches so that they don't see us. As a woman, once your life misses the track, things are difficult… After corona[virus] they are​ now collecting and taking people away en masse. They take them​ to the police station and give corrective advice… The police… abuse us a lot… they force us to have intercourse with them… Yes. This is our life.​

However, key informants noted a lack of government action in responding to these heightened risks. For example, a district official from a Women and Children Affairs office in Ethiopia acknowledged the recent spike in age‐ and gender‐based violence at home and on the streets but explained that they were unable to act due to the closure of local courts.

2. Inadequate delivery of essential services – education, health, psychosocial support

Adolescents also highlighted a second dimension of structural violence, namely inadequate delivery of essential services following public health measures to stem the spread of the virus. For example, they emphasised that while distance education services had been established by the government, existing barriers prevented many young people accessing these. In Bangladesh, to mitigate the loss of classroom teaching, the government established television‐based classes for National Curriculum students up to Grade 10 broadcast in different time slots. While TV lessons were also uploaded onto YouTube and Facebook, most adolescents in our sample were not able to access them due to technological divides. As a 14‐year‐old girl in Rupnagar noted: *“In our village we do not have any TV. So I can't watch the TV classes”*. Intermittent internet connection and a lack of mobile phones also limits access to such platforms. An 18‐year‐old boy explained:

My problem is that I have no Wi‐Fi in my house. So I need to purchase the internet and therefore I miss classes sometimes… It takes a lot of bandwidth for that 1‐hour class.

In Ethiopia, inequalities in access were starker still due to overall lower levels of connectivity. As an education bureau official in South Gondar explained:

How many people have TV and radio? How many of the students in grades 11 and 12 have access to laptops and read things by saving on memory sticks? Even for us, while sitting in the woreda [town], for how many days do we have access to electricity and water?… In such circumstances, ‘education on television and radio’ is unthinkable.

Students across the research sites highlighted that they had had no interaction with their teachers, many of whom had moved away from the area. Low levels of parental literacy also meant they had very limited support for studying at home. As a 16‐year‐old girl from a rural community noted: “*We do not have contact with our teachers. Teachers also consider it as vacation, and they went [back to their home town]. No one asks or sends us questions”*.

The dearth of accessible education services emerged as especially challenging for adolescents with disabilities, as educational facilities had been one of their few sources of socialising regularly. As a 19‐year‐old girl with a physical disability highlighted: “*School was a place where I interacted with people and spent happy time”*. For many young people with disabilities, however, lockdowns and closure of educational institutions means they no longer have contact with peers, which some described as very stressful, and inducing symptoms of depression.

Even pre‐pandemic, Rohingya adolescents were not permitted to attend formal education and were restricted to non‐formal education services through camp‐based learning centres, with older adolescents often excluded as the curriculum focuses on younger children. UNICEF and the Bangladesh government had planned to introduce the Myanmar curriculum to upper‐level students in 2020, but this was halted due to the pandemic. Adolescents expressed sadness at learning centres closing and the lack of alternatives during lockdown. A 15‐year‐old boy, who was enrolled in religious education (*madrasa*) said, “*Since the mosque‐madrasa cannot be opened, as the government has closed it, I am not able to study [now]. That's why a lot of sadness is in my mind”*. For adolescents in skills training programmes, the lockdown similarly threatened opportunities for future livelihoods that they and their families so urgently need. As one 15‐year‐old girl explained:

Now I can't go to school. Before corona[virus], I learnt tailoring. [Now] I can't go out, so I have to stay home, can't learn the work. Before, I had the income and at least I could manage the expenditure of daily shopping. But now, I can't go out. I have no brother, no father. What will I do? So the government's actions [are] good for the disease. But it isn't good for securing food and for our survival.

Turning to health services, adolescents in Dhaka, Bangladesh, highlighted the very limited testing facilities and inadequate access to treatment for people with the virus. Some young people emphasised the stigma and fear attached to COVID‐19, noting that some people were hiding their symptoms for fear of being taken away by the police and kept in isolation. A 17‐year‐old girl claimed, “*The people in our area are panicking. If someone has a slight fever, no one goes near them. Everyone stays in fear”*. Many adolescents also mentioned increased difficulties in receiving non‐COVID‐19 health services, with doctors not at work or hastily diagnosing patients and sending them away. Similarly, in Cox's Bazar, Rohingya adolescents also reported self‐excluding from services based on fear of contracting COVID‐19. A 17‐year‐old boy explained:

We cannot go to the hospital if we are sick. Earlier, we used to go to the IOM [International Organization for Migration] hospital, but now we are not allowed to go there. Now when someone goes there, they say that he/she has contracted the coronavirus. That's why people are not willing to go to the hospital.

Challenges in accessing essential health services were also a common theme across communities in Ethiopia. As a 17‐year‐old married girl from a rural community noted:

*The health extension worker has not come since the disease outbreak. She locked down her office and stays put. She does not give any service and stopped her previous activities*.

Other adolescents, including those in towns, concurred. As a 19‐year‐old girl with a visual impairment in South Gondar explained:

There is a hospital here. I don't know why, but they are refusing to accept patients… They are refusing to accept patients with other sickness… What can I do? If it is my fate I try not to pass it onto others. I will die alone.

Key informants confirmed these challenges, highlighting concerns about access to sexual and reproductive health services, including maternity care, for adolescent girls and young women. As a health official in a pastoralist community in Afar emphasised:

… women are suffering due to childbirth and in our locality are dying due to excessive bleeding… Only in two days, three young women died due to childbirth.

Respondents also explained that limited access to health services during the pandemic meant that other infectious diseases were being left to spread unchecked, including measles in East Hararghe and South Gondar, and Chikungunya disease in Dire Dawa.

In terms of mental health services, adolescents highlighted high levels of mental distress and social isolation due to school closures, unemployment, and heightened intra‐household tensions, noting the dearth of support services – either governmental or non‐governmental. As a migrant girl living in a large urban centre explained: “*There are no jobs and school is now closed and I don't have anywhere to go. The church is closed, which means we only sit at home. We are so stressed”*. While mental health services in Ethiopia were generally very limited before COVID‐19, adolescents and key informants alike underscored that the situation had been exacerbated. Lockdown measures mean that many service providers are home working, and there is consequently a dearth of opportunities for young people to interact with peers. As a Women and Children Affairs official from Afar explained:

*As you know schools are closed, and there is no adolescent recreation centre or wider space for adolescents to spend their time… Adolescents risk getting depressed due to the lockdown…*

3. Failure to provide adequate livelihood opportunities and social protection support

A third key domain of government and humanitarian agency relative invisibility during the pandemic has been manifest in the failure to protect the livelihoods of young people and their families, including very limited provisioning of social protection support. In Dhaka, a significant number of adolescents reported not receiving any food, relief, or cash aid from the government or NGOs as targeted beneficiaries are the “extreme poor”, while many urban slum‐dwellers are deemed “low income” and have fallen through the cracks during the crisis. A 17‐year‐old girl whose parents own a small shop and live in their own house underscored such challenges:

Yes, they are distributing these things [relief] but only to those people who are very *needy and helpless, such as the rickshaw‐pullers or the beggars… but they don't give anything to people like us*.

Similarly, some families involved in small businesses and day labour have experienced interruptions to their livelihoods as they have been working only intermittently under police surveillance. As a 17‐year‐old girl noted:

My parents close the shop [roadside fruit shop] whenever the police come. The police come every other day. So, whenever the police come, people hurriedly shut down everything and run to their home.

The wide lockdown, the ensuing financial instability, and the failure to provide timely social protection forced some families to leave the capital and go back to their home villages to better cope with expenses and rising food prices. As a 14‐year‐old girl noted:

My mother worked as a domestic helper but during this lockdown she lost her job. Now we are facing a financial crisis. But still we have to pay the house rent. So my parents decided to go back to the village.

Rohingya refugees face a particularly precarious economic situation. Already banned from engaging in formal work, males are no longer able to support the household through informal economic activities, given bans on mobility outside the camp. As a 15‐year‐old married girl explained: *“People get less rations now… People also can't go out to find work. If we have some help from government we can live in a proper way and can get enough food”*. A 16‐year‐old girl added: *“They [the army] come here every day… Many people were fined as they opened their shops”*. Our data showcases that the loss of paid work is a major worry for adolescents, as an 18‐year‐old boy explained, “*We could earn money then. Now we can't. My elder brother used to work [for an NGO]… now he can't… Depression comes as we can't earn money now”*.

Adolescents in Ethiopia reported a similarly precarious situation. As a 19‐year‐old girl from a large urban centre noted, meeting basic subsistence needs had become very challenging:

People in the developed world can afford to stay at home for a long time as they have income which enables them to stay without earning for five days or more… But in our case it is so hard to do that. For instance, my monthly earning was 700 birr [$20] and my mother used to earn 200 birr [$5.70] per day… I am afraid that people could die of starvation if the stay‐at‐home order is implemented for an extended period… There are many households who are even poorer than ourselves.

These vulnerabilities were especially acute among young people with disabilities, many of whom were dependent on relatives and neighbours for charitable support, which was a cause of great stress. As a 19‐year‐old with a physical disability noted:

Since COVID, I have been leading a hand‐to‐mouth life. I have no job. I depend on some money that my friends give me. This cannot help me to lead a stable life. Before COVID, I served in a local church and some people had given me some money as alms, but with the closure of the church, everything has been stopped and I face a shortage of food every day. It is miserable for me to lead such a life now.

As an official from the Bureau of Labour and Social Affairs noted: “Because when workers were fired from different industries [at the onset of the pandemic] they lost their main source of income and then they could not have money for paying the house rent and for food let alone protecting themselves from COVID by using preventive supplies​ which they could not afford. So, they were exposed to street life”.

## 6 DISCUSSION

This paper has sought to explore how states can protect vulnerable young citizens and deliver on assistance in the context of an emergency such as COVID‐19 and the implications for legitimacy when they fail to do so. The research presented shows that measures taken by states to prevent the spread of COVID‐19, such as closure of schools, basic services, and closure of certain employment sectors, have had a major negative impact on young people. The toll has been greatest for those who are already among the most marginalised, such as the extreme urban poor, migrant workers, young people with disabilities, and refugee and internally displaced youth. A structural violence perspective highlights how intersecting disadvantages facing certain population groups mediate interactions with the state and therefore access to assistance. In taking measures to prevent the spread of COVID‐19, states must navigate the protection of citizens’ health on the one hand, and their human rights and livelihoods on the other. Yet in Bangladesh and Ethiopia, the lines between these obligations have been blurred. The result is the state has become largely “invisible” as a protection actor – instead only making itself visible through structural violence.

Writing on the role of social protection in relation to COVID‐19, Razavi et al. (2020) warn that the pandemic is throwing existing social inequalities into sharp relief, risking further undermining the social contract in unstable contexts; but equally, the importance of social protection has gained new public and political awareness as a means of “buffering” the risks the pandemic presents to the most marginalised. The fundamental ethos of social protection is the anticipation of shocks that may exacerbate vulnerabilities. As such, it is essential that systems are in place to respond immediately when a crisis occurs. Our findings as to vulnerable adolescent experiences of the COVID‐19 pandemic has illuminated gaps within social protection systems within LMICs. Yet where states are willing to expand these systems to respond to the pandemic, the slow pace of support by international aid actors presents challenges in doing so. While the United Nations system has sought to catalyse existing extensions of national social protection schemes, and visible engagement at the design stage, there is little evidence that these discussions are being translated into the urgent action necessary to support vulnerable groups in either Bangladesh or Ethiopia.

Our findings also show that young people have not been specifically targeted within social protection programming responses during the pandemic in either country. These programmes have traditionally targeted aggregate households rather than individuals. Yet in reality, most young people in urban areas of Ethiopia who have migrated from rural areas for work and have thus been put at risk of destitution by the shutdown of factories and construction sites are there alone.

Evidence from refugee and migrant communities in both contexts shows that states are also not doing enough to honour their commitments under the Global Compact on Refugees. One of the Compact's central tenets is the inclusion of refugees, internally displaced people, and stateless people in national health care systems – even more vital given COVID‐19. UNHCR (2020b) has called for states to scale up services and support for the most vulnerable groups, including children and youth, as part of the COVID‐19 response. Ensuring that the most marginalised populations are not “left behind” lies at the centre of the 2030 Agenda. Yet the experiences of adolescent Rohingya refugees in Cox's Bazar and IDPs in Ethiopia indicates serious shortcomings in the state's ability to deliver on these responsibilities.

Finally, analysis of power relations does not end at the relationship between states and citizens; it demands attention to the role of the wider international community in upholding the 2030 Agenda during this crisis.

## 7 CONCLUSION

This paper has sought to understand the socioeconomic impact of COVID‐19 on adolescents and youth in lower‐ and middle‐income countries (LMICs) who have migrated for work, are among the urban poor, or have been forcibly displaced. The paper finds that the vulnerability of disadvantaged adolescents in Bangladesh and Ethiopia has been intensified during the COVID‐19 pandemic due to the relative “invisibility” of the state as an agent of protection in both contexts. Age‐related power inequalities that lead to young people's marginalisation is combined with structural violence whereby young people are not only deprived of the ability to meet their basic needs but also placed at heightened risk of various forms of violence. These forms include interpersonal violence within the household, by police, and within the community due to growing economic stressors, closure of services, and inadequate surveillance and reporting mechanisms that are accessible to adolescents.

Our focus on power relations at multiple levels reveals that the interdependent social and economic systems in which adolescents are situated shape not just vulnerabilities but also can constrain opportunities for effective intervention. The invisibility of the state as a protective actor in a time of crisis equates to complicity in structural violence. State legitimacy must be linked to the state's willingness to protect its most vulnerable citizens, as outlined in the transformational governance strategy within the SDGs’ leave no one behind agenda.

## 8 RECOMMENDATIONS

The invisibility of adolescents within social protection measures, despite evidence of their heightened vulnerability in relation to the impact of COVID‐19, underlines the need for age‐tailored policy design and programme delivery. Expanding and investing in social protection systems in LMICs so that they can be rapidly mobilised during crises is vital to addressing the pre‐existing inequalities which have intensified the compounded disadvantages and forms of structural and physical violence experienced by the most vulnerable young people in Bangladesh and Ethiopia.

Rather than making assumptions about young people's networks and relationships, social protection programming must start from the realities of what resources and assets are available to young people. Adolescents’ capacities to draw on networks and relationships in ways that keep them safe is also shaped by gender, nationality status, place of residence, and their relationships with the wider community. These networks may be supportive of young people, as seen in the case of adolescents with disabilities who have been able to turn to relatives for protection and assistance during the pandemic. Yet they may also render them more vulnerable; for example, married adolescent girls who find themselves restricted to the home to ostensibly keep them safe but find themselves socially isolated and at risk of intimate partner violence.

It is important to note that there is likely to be a growing need for social protection mechanisms to be quickly mobilised during future crises, particularly in the context of climate‐related shocks and rising levels of global displacement. Social protection is a basic human right. SDG 1.3 promotes the establishment of social protection systems for all as being key to eradicating poverty. Structural inequalities shape how interventions are experienced; if these dynamics are taken into account, effective social protection has the potential to promote transformative social outcomes and justice for marginalised groups (Sabates‐Wheeler and Devereux 2008; Holmes et al. 2011). An essential part of confronting and overturning colonial legacies of health inequalities is assisting countries to prepare for future crises through addressing obstacles to pro‐poor and inclusive growth and development that would enable states to generate more resources for progressive social protection programming (Farmer et al. 2006; Hickel, 2017b).

Social protection interventions such as cash‐plus programmes (combining transfers with linkages to complementary services and support) can help to mitigate the vulnerabilities presented by a crisis such as COVID‐19. Yet despite the existence of platforms for social protection targeting the most vulnerable citizens in Bangladesh and Ethiopia, this article has shown that there is limited evidence of the state in either context mobilising existing social protection platforms to assist the growing number of young people who have been made even more vulnerable by the pandemic. Our primary recommendation is that these platforms are rapidly mobilised and expanded, informed by vulnerability assessments that are context‐specific and reflect age‐, gender‐, and intersectional concerns, and with adequate funding from national governments and the international community to provide at scale and sustained rather than emergency‐only forms of support.

## CONFLICT OF INTEREST

The authors report no conflict of interest in relation to this research.

### PEER REVIEW

The peer review history for this article is available at https://publons.com/publon/10.1111/issj.12358

## Data Availability

The author has provided the required Data Availability Statement, and if applicable, included functional and accurate links to said data therein.
